# Three-dimensional morphological study of type B lateral malleolar fractures with special reference to the end-tip location of proximal apexes

**DOI:** 10.3389/fbioe.2023.1152775

**Published:** 2023-05-04

**Authors:** Wei-Bin Wang, Shi-Min Chang

**Affiliations:** ^1^ Department of Orthopedic Surgery, Yangpu Hospital, School of Medicine, Tongji University, Shanghai, China; ^2^ Department of Orthopedic Trauma, Ningbo No. 2 Hospital, Ningbo, China

**Keywords:** ankle fracture, lateral malleolar fracture, type B fracture, end-tip apex, spike, fracture line map

## Abstract

**Objective:** We aimed to describe the morphological characteristics of Danis–Weber type B lateral malleolar fractures, with special attention given to the end-tip locations of fracture apexes, and to construct a 3D (three-dimensional) fracture line map.

**Methods:** A total of 114 surgically treated cases of type B lateral malleolar fractures were retrospectively reviewed. The baseline data were collected, and computed tomography data were reconstructed in a 3D model. We measured the morphological characteristics and the end-tip location of the fracture apex on the 3D model. All the fracture lines were superimposed on a template fibula to generate a 3D fracture line map.

**Results:** Among these 114 cases, 21 were isolated lateral malleolar fractures, 29 were bimalleolar fractures, and 64 were trimalleolar fractures. All the type B lateral malleolar fractures demonstrated a spiral or oblique fracture line. As measured from the distal tibial articular line, the fracture started at −6.22 ± 4.62 mm anteriorly and terminated at 27.23 ± 12.32 mm posteriorly, and the average fracture height was 33.45 ± 11.89 mm. The fracture line inclination angle was 56.85° ± 9.58°, and the total fracture spiral angle was 269.81° ± 37.09°, with fracture spikes of 156.20° ± 24.04°. The proximal end-tip location of the fracture apex was classified into four zones in the circumferential cortex: zone I (lateral ridge) in seven cases (6.1%), zone II (posterolateral surface) in 65 cases (57%), zone III (posterior ridge) in 39 cases (34.2%), and zone IV (medial surface) in three cases (2.6%). Altogether, 43% (49 cases) of fracture apexes were not distributed on the posterolateral surface of the fibula, as 34.2% (39 cases) were located on the posterior ridge (zone III). The aforementioned morphological parameters in fractures with zone III, sharp spikes, and further broken spikes were greater than those in zone II, blunt spikes, and fractures without further broken spikes. The 3D fracture map suggested that the fracture lines with the zone-III apex were steeper and longer than those with the zone-II apex.

**Conclusion:** Nearly half of type B lateral malleolar fractures had their proximal end-tip of apexes not on the posterolateral surface, which may impair the mechanical application of antiglide plates. A steeper fracture line and longer fracture spike indicate a more posteromedial distribution of the fracture end-tip apex.

## 1 Introduction

The Danis–Weber classification divides ankle fractures into three types—type A, type B, and type C—according to the relationship between the lateral malleolar fracture line and the tibiofibular syndesmosis (Fonseca et al., 2017). Type B fractures account for approximately 66.2% of all ankle fractures, and the incidence increases with aging (Juto et al., 2018). Type B fractures account for up to 77% of surgically treated ankle fractures (Vieira Cardoso et al., 2021).

Various techniques have been used to fix type B lateral malleolar fractures, including plate osteosynthesis, intramedullary nails, Kirschner wire tension bands, and interfragmentary lag screws (Pearce et al., 2020). Plate osteosynthesis is still the most commonly used construct (Hsu et al., 2019). According to the applied position and biomechanical function, plates are used in a lateral neutralization mode or posterolateral antiglide mode. Biomechanical studies have shown that posterolateral antiglide plate fixation has more robust mechanical stability (Schaffer and Manoli, 1987; Minihane et al., 2006; Buscharino et al., 2013; Deng et al., 2022). The posterolateral antiglide plate also has the advantages of less skin irritation, fewer wound complications, and a lower reoperation rate (Winkler et al., 1990; Kilian et al., 2017; Wenger et al., 2021). Moreover, the proper application of low-profile plates does not lead to an increased incidence of peroneal tendonitis (Weber and Krause, 2005; DeKeyser et al., 2022).

The key to the application of the posterolateral antiglide plate in type B lateral malleolar fractures is to compress over the end-tip apex of the distal fragment by an undercontoured plate. However, not all type B lateral malleolar fracture apexes are located on the posterolateral surface of the fibula, which may impair the effect of antiglide mechanisms (DeKeyser et al., 2022). To the best of our knowledge, no previous study has examined the distribution of fracture apexes of type B lateral malleolar fractures or their relationship to the fracture morphology. Therefore, the purpose of this study is to describe the morphological characteristics of type B lateral malleolar fractures, explore their relationship with the locations of the fracture apexes, and construct a 3D fracture line map to more clearly reveal these relations.

## 2 Methods

### 2.1 Patient selection and data collection

All procedures were approved by our hospital’s Institutional Review Board. Informed consent was waived because of the retrospective nature of the study. All patients with ankle fractures, who were surgically treated in our hospital from 1 January 2017 to 31 May 2022, were reviewed. The inclusion criteria were as follows: 1) Danis–Weber type B ankle fracture, 2) preoperative X-ray and computed tomography (CT) taken in our hospital, 3) age ≥18 years, and 4) no previous fracture of the ankle joint. The exclusion criteria were as follows: 1) Danis–Weber types A and C fractures, 2) pathological fractures, 3) obvious deformities of the ankle, 4) severely comminuted fractures, and 5) poor quality of CT image data (slice thickness >1 mm, incomplete scanned image, *etc.*). All CT examinations were performed using conventional cross-sectional scans and 64-detector row CT scanners (Siemens Somatom Sensation, Germany). The scanning conditions were as follows: voltage 120 kV, current 90 mA, layer thickness 0.75 mm, and reconstruction matrix 512 × 512. The scan plane was parallel to the cross-section of the tibia.

Demographic data, including age, sex, and injury side, were recorded. According to the extension of the fracture on CT, they were divided into single lateral malleolar fractures, lateral malleolus combined with medial malleolar fractures (bimalleolar M), lateral malleolus combined with posterior malleolar fractures (bimalleolar P), and trimalleolar fractures.

### 2.2 Comparison of the posterolateral antiglide plate and lateral neutral plate

**FIGURE 1:**
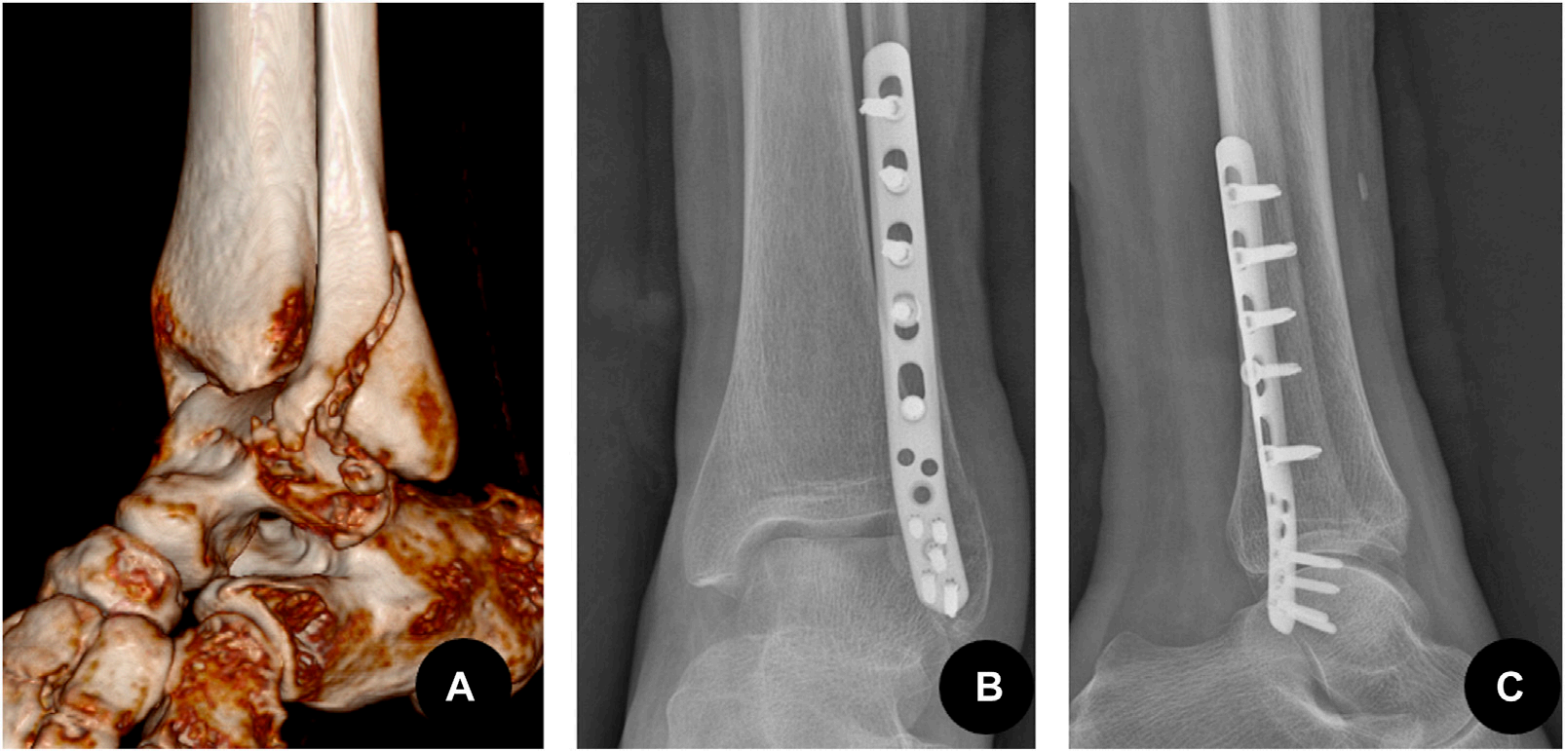
Type B lateral malleolar fracture fixed by the antiglide-buttress plate mode that is applied on the posterolateral side. **(A)** A 72-year-old female patient with injury to the left lateral malleolus. Preoperative CT three-dimensional reconstruction showed a type B spiral fracture, the distal fragment moved backward and upward. **(B)** The postoperative anteroposterior X-ray showed that the fracture of the lateral malleolus was fixed by the posterolateral anti-glide plate. **(C)** Postoperative lateral X-ray.

**FIGURE 2:**
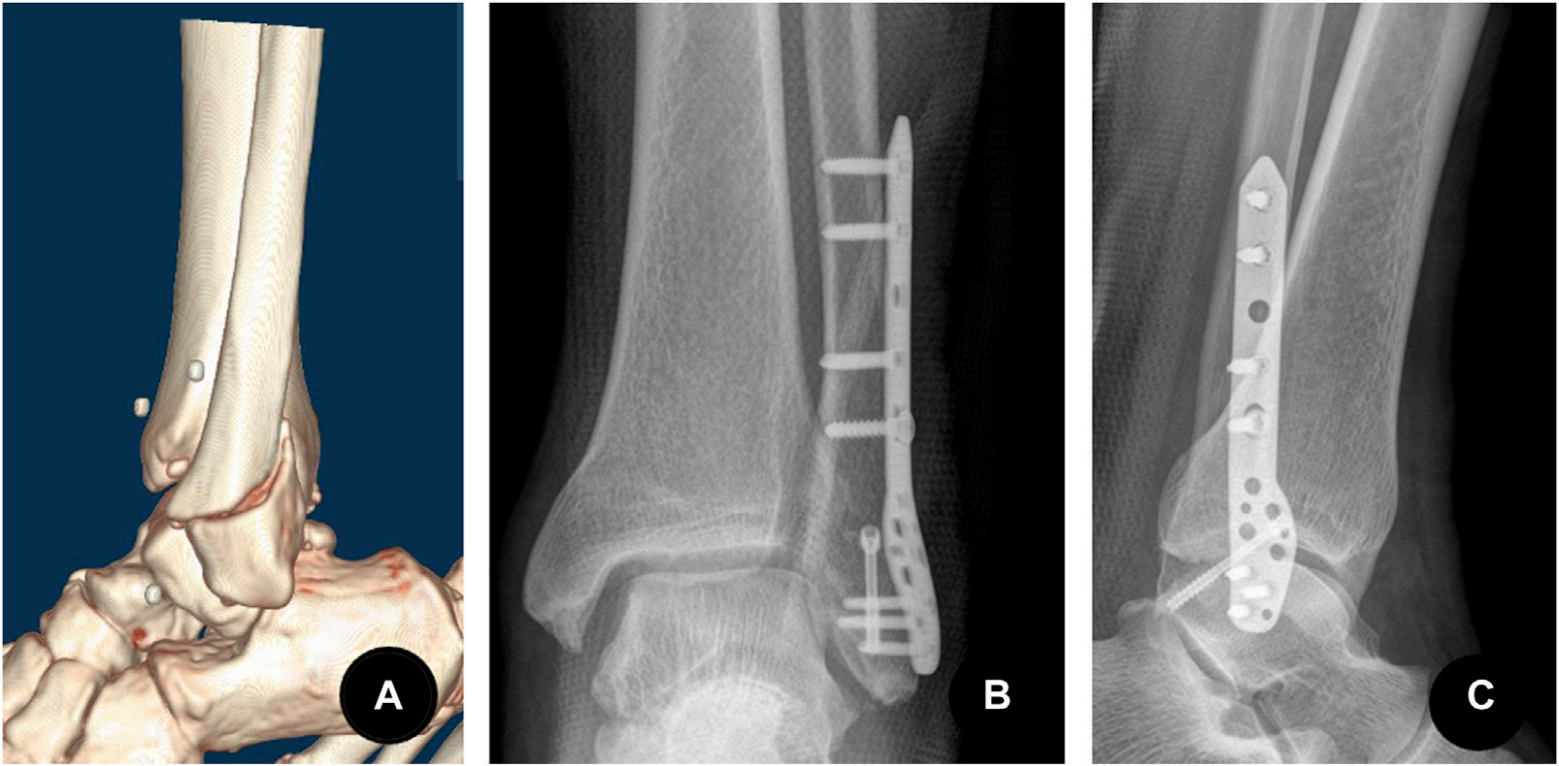
Type B lateral malleolar fracture fixed by a compression screw and a neutral plate mode that is applied on the lateral side. **(A)** A 57-year-old male patient with injury to the left lateral malleolus. Preoperative CT three-dimensional reconstruction showed a type B spiral fracture of the lateral malleolus, the distal fragment moved backward and upward. **(B)** The postoperative anteroposterior X-ray showed the lateral malleolus was fixed with a compression screw crossing the fracture lines and a lateral plate. The lateral plate acted as a neutralizing plate to protect the compression screw.**(C)** Postoperative lateral X-ray.

Clinically, type B lateral malleolus fractures are commonly fixed with posterolateral antiglide plates or lateral neutral plates. The different features between them are shown in Table 1. An illustrative case of the posterolateral antiglide plate is shown in Figure 1, and a case of the lateral neutral plate is shown in Figure 2.

**TABLE 1:** Comparison of the posterolateral antiglide plate and lateral neutral plate.

	Posterolateral antiglide plate	Lateral neutralization plate
Plate position	Posterolateral surface of the distal fibula	Lateral surface of the distal fibula
Plate function	Counteracts the shifting tendency of the distal fragment	Neutralizes the force subjected by the compression screw crossing the fracture line
Advantages	1. When using the same plate, the biomechanical strength is higher than that of the lateral plate	1. There are more distal screws on the lateral plate, and different screw implantation positions can be selected according to the fracture morphology
	2. Type B lateral malleolus fractures are often combined with posterior malleolus fractures. It is more convenient to apply a posterolateral plate with less soft tissue stripping when treating through a posterolateral incision	2. If syndesmotic separation co-exists at the same time, syndesmotic screws can be placed through the lateral plate directly
	3. The posterolateral plate is located on the posterolateral side of the distal fibula with more soft tissue coverage and less stimulation to the lateral skin	3. The lateral plate would not stimulate the peroneus longus and brevis tendon
	4. The distal screw of the posterolateral plate can be fixed with double cortex, the fixation strength is high and the risk of entering the joint is low	
Disadvantages	1. Inappropriate placement of the plate or the distal screws may irritate the peroneus longus and brevis tendons	1. The plate may irritate the lateral skin causing lateral ankle pain
		2. The distal screws can only be fixed with single cortex and may loosen in osteoporotic patients
		3. Inappropriate placement of the distal screws may enter the joint cavity

### 2.3 Morphological measurement

**FIGURE 3:**
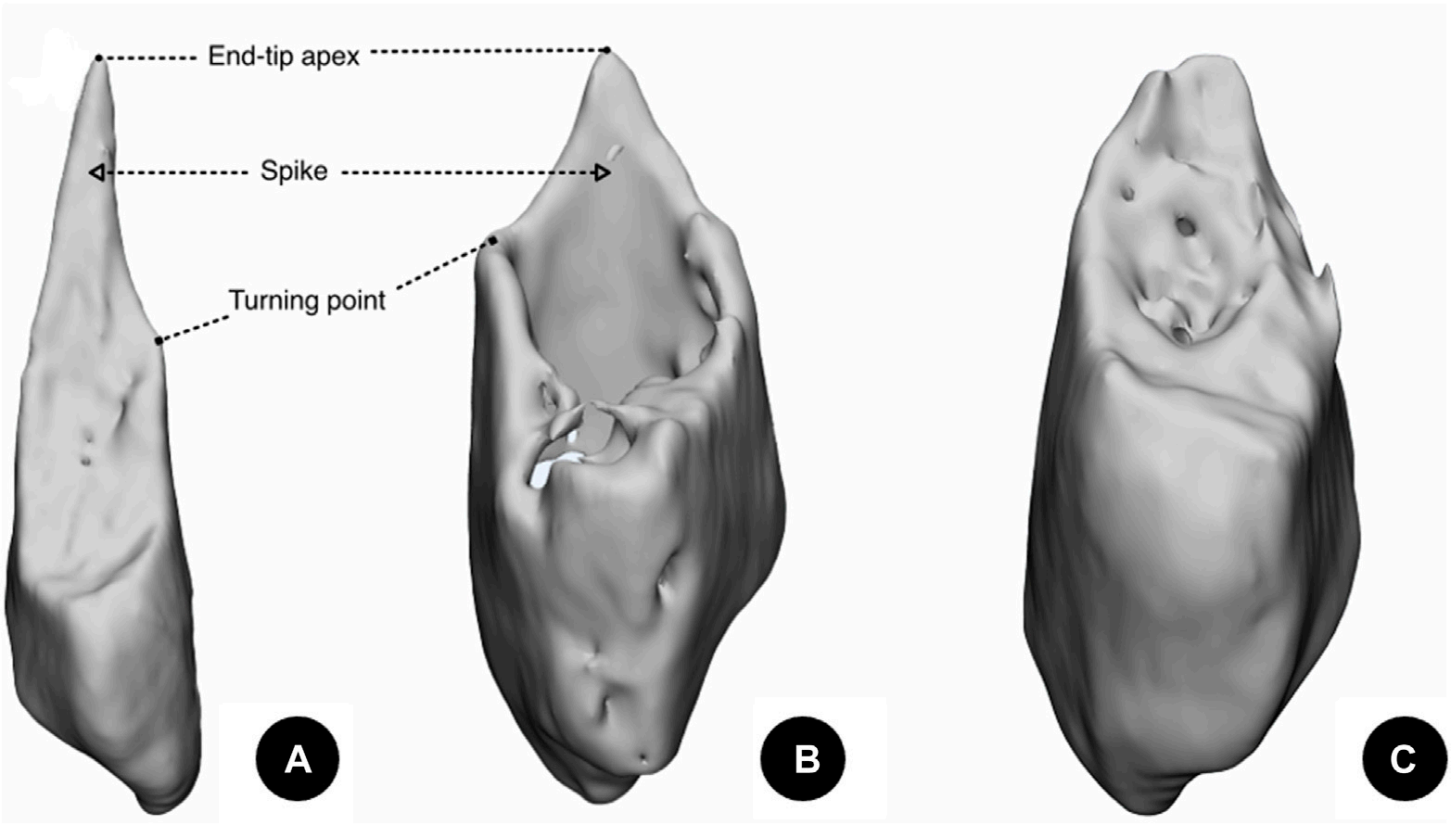
Different patterns of type B lateral malleolar fractures. **(A)** Fracture with a long sharp spike. **(B)** Fracture with a short sharp spike. **(C)** Fracture with a blunt spike.

**FIGURE 4:**
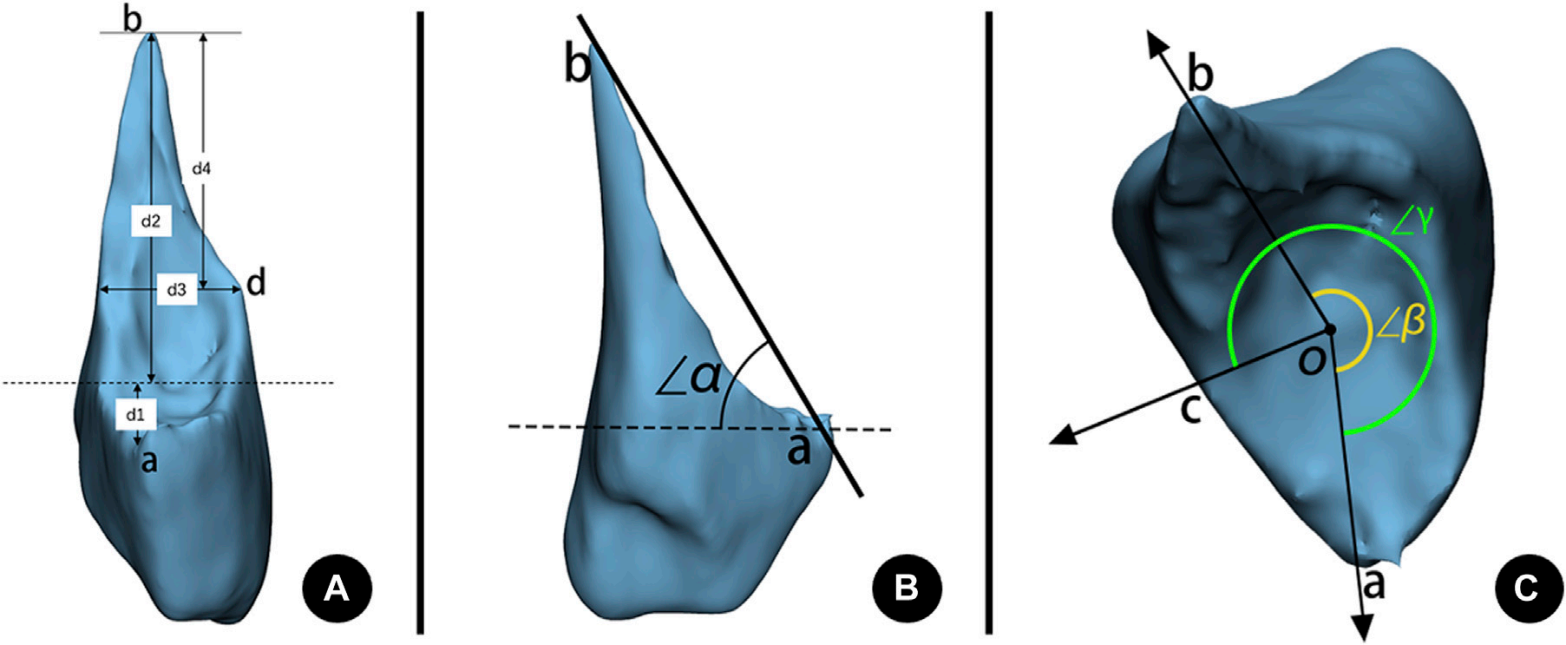
Morphological measurements of a lateral malleolar fracture. **(A)** Fracture height measurement. d1: Anterior fracture height. d2: Posterior fracture height. d3: Base width of a sharp spike. d4: Height of a sharp spike. **(B)** Fracture inclination angle measurement. ∠α: Inclination angle of the fracture. **(C)** Fracture spiral angle measurement. ∠β: Spiral angle of the spike. ∠γ: Spiral angle of the total fracture. a: The lowest point of the spiral fracture line starting on anterior. b: The end-tip point of the fracture apex. c: The terminal point of the spiral fracture line. d: Turning point on the ascending curve of the fracture line. o: The fibula circumcircle center on the plane of the distal tibia’s articular surface. Dashed line: The plane of the distal tibia’s articular surface.

According to whether there was a turning point on the ascending curve of the fracture line in the 3D images, the fractures were divided into two patterns: fractures with a sharp spike and fractures with a blunt spike. In addition, the height and base width of the sharp spikes were measured, and if the ratio of height to width was ≥1, it was further defined as a long sharp spike pattern, and if the ratio was <1, it was defined as a short sharp spike pattern (Figure 3; Figure 4). A total of eight fracture morphological parameters, including lengths and angles, were selected for measurement (Table 2). The spiral angles of the whole fracture and the spike were measured according to the fibula’s rotation point, which was set on the fibula circumcircle center (o) on the plane of the distal tibial plafond level.

**TABLE 2:** Morphologic measurements of type B lateral malleolar fractures.

	Morphologic index	Definition
1.	Anterior fracture height (d1)	Vertical distance from the lowest point of the fracture line to the plane of the distal tibial articular surface (below the articular surface is a negative value)
2.	Posterior fracture height (d2)	Vertical distance from the end-tip point of the fracture apex to the plane of the distal tibial articular surface
3.	Vertical fracture height (d2-d1)	Vertical distance from the lowest point of the fracture line to the end-tip point of the fracture apex
4.	Fracture inclination angle (∠α)	The angle of the line of the lowest point of the fracture line and the end-tip point of the fracture apex to the plane of the distal tibial articular surface
5.	Spiral angle of the fracture (∠γ)	The angle of the lowest point of the fracture line externally rotates to the terminal point of the spiral fracture
6.	Spiral angle of the spike (∠β)	The angle of the lowest point of the fracture line externally rotates to the end-tip point of the fracture apex
7.	Height of the sharp spike (d4)	Vertical distance from the end-tip point of the fracture apex to the turning point on the ascending curve of the fracture line
8.	Base width of the sharp spike (d3)	Distal fragment width at the plane of the turning point on the ascending curve of the fracture line, which is parallel to the distal tibial articular surface

### 2.4 The distribution of fracture end-tip apexes

**FIGURE 5:**
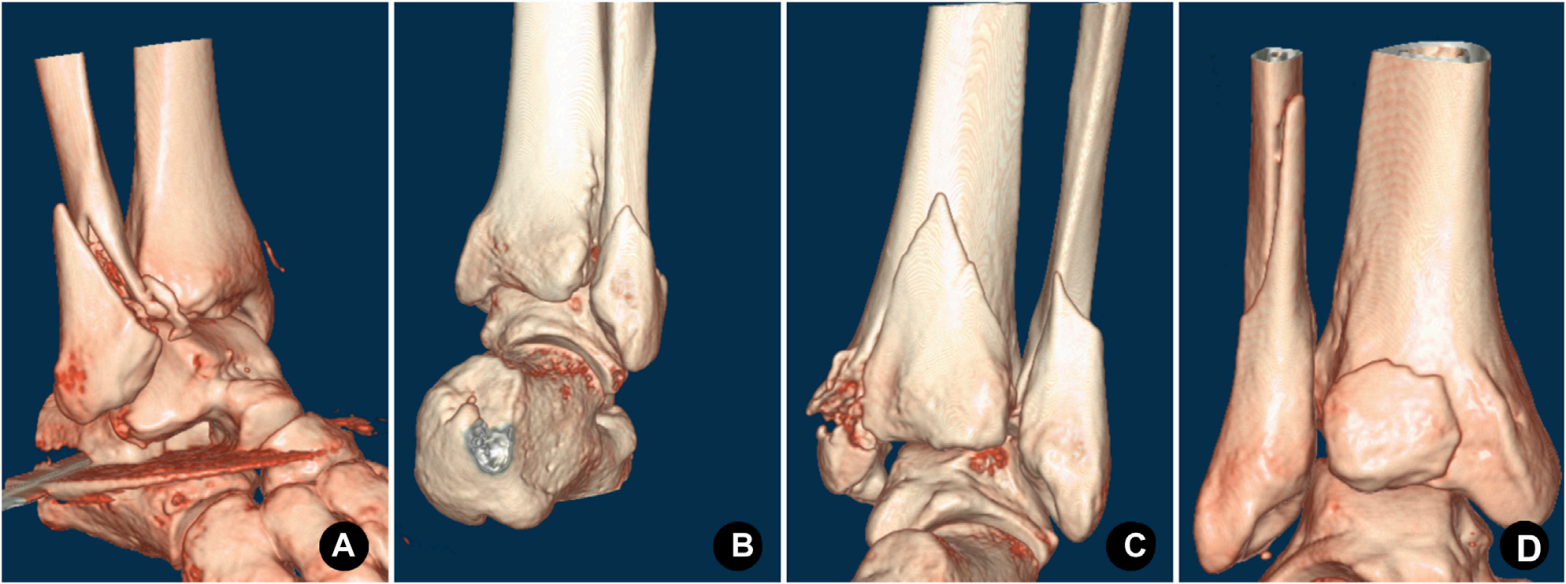
Distribution of the end-tip apex was classified into four zones in 3D-CT images. **(A)** Zone I, the apex located on the lateral ridge. **(B)** Zone II, the apex located on the posterolateral surface. **(C)** Zone III, the apex located on the posterior ridge. **(D)** Zone IV, the apex located on the medial surface.

The locations of fracture end-tip apexes were observed on the 3D model and were divided into four zones (Figure 5): zone I (the lateral ridge, including the 1 mm range inside and outside the midline of the ridge), zone II (the posterolateral surface), zone III (the posterior ridge, including the 1 mm range inside and outside the midline of the ridge), and zone IV (the medial surface). If the fracture spike was broken, the corresponding position on the proximal bone fragment (spike gap) was recorded.

### 2.5 Mapping of fracture lines

**FIGURE 6:**
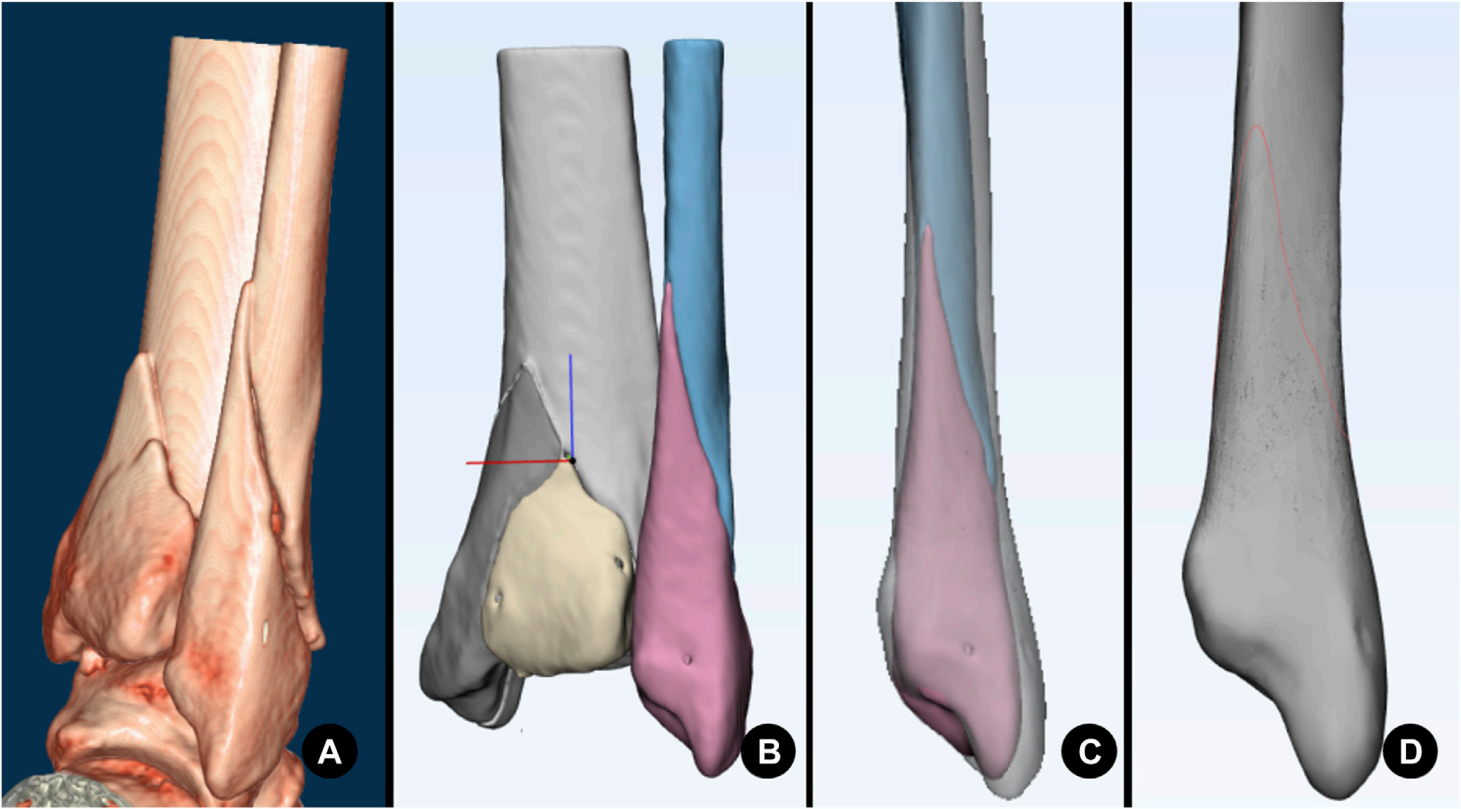
A series of images illustrating the progression of mapping. **(A)** CT volume rendering. **(B)** Surface reconstruction and fracture reduction. **(C)** Fitting to the template fibula. **(D)** Drawing the fracture line on the template (red curve).

Referring to the fracture line map technology of Zhang et al. (2020), the CT scan data of the patient were imported into Mimics 21.0 (Materialise N.V., Belgium) in the DICOM format. We select the bone cortex threshold and segment the 3D model of the fibula and tibia so that each bone fragment has independent attributes. Then, all of them were imported into 3-matic 13.0 (Materialise N.V., Belgium), and the fracture was virtually reduced through rotation and translation. Then, the file was saved in the form of MXP by the name of the patient. All CT data, including the data of a 43-year-old male patient’s right ankle without apparent deformity, which was used as the standard template for mapping, were collected as previously mentioned. Importing the standard template into 3-matic, the data of other patients were imported one by one. First, the template was set as medium transparency. Second, after fitting the reduced lateral malleolar fracture to the template with reference to anatomical landmarks such as the fibula fossa, the anterior tuberosity of the fibula, and the posterolateral surface of the fibula, the fracture line was drawn on the template with reference to the reduced fracture and grouped by the location of the end-tip apex. All fracture lines were shown on the template to generate the overall fracture line map and shown separately by groups to present the maps for zone I, zone II, zone III, and zone IV fractures (Figure 6).

### 2.6 Statistical analysis

Categorical data were summarized using frequencies and percentages, and the chi-squared test was used for comparisons between groups. Continuous variables were calculated as the mean and standard deviation. The Shapiro‒Wilk test and Levene’s test were used to judge whether the data conformed to a normal distribution and homogeneity of variance. A *t*-test was used to compare normally distributed variables, while the Mann‒Whitney *U* test was used to compare non-normally distributed variables. SPSS 26.0 (IBM, United States of America) was used for statistical analysis, and all tests were two-way tests with a test level of α = 0.05.

## 3 Results

A total of 230 surgically treated ankle fractures were reviewed, of which 149 (64.9%) were type B lateral malleolar fractures. According to the inclusion and exclusion criteria, 114 patients were ultimately included in the study, with a mean age of 59.2 ± 14.0 years (range 20–89 years). There were 46 males (40.4%) with a mean age of 54.0 ± 14.5 years (range 20–76 years) and 68 females (59.6%) with a mean age of 62.5 ± 12.9 years (range 28–89 years). Among the female patients, 48 (70.6%) were ≥60 years old. Of the 64 patients (56.1%) with trimalleolar fractures, 43 (67.2%) were female.

### 3.1 Morphological characteristics of type B lateral malleolar fractures

Overall, the average anterior fracture starting point was −6.22 ± 4.62 mm from the distal tibial articular line, and the posterior terminating point was 27.23 ± 12.32 mm, with a total fracture vertical height of 33.45 ± 11.89 mm. The fracture inclination angle was 56.85° ± 9.58°. The total fracture spiral angle was 269.81° ± 37.09°, including a fracture spike of 156.20° ± 24.04°. These parameters in the zone III fracture apex, such as the fracture height in the anterior, posterior, and total directions, the fracture spiral angle, and the spike spiral angle, were greater than those in the zone II fracture apex (Table 3).

**TABLE 3:** Comparison between type B lateral malleolar fractures with different apex distributions.

	Fracture apex distribution				Total	*p*-value
	Zone I	Zone II	Zone III	Zone IV		
No. of cases (%)	7 (6.1%)	65 (57%)	39 (34.2%)	3 (2.6%)	114 (100%)	
Patient age (yr)^a^	63.1 ± 9.3	63.6 ± 12.0	52.7 ± 14.3	34.3 ± 7.1	59.1 ± 14.1	0.000^b^
Sex^c^						0.719^d^
Male	2	26	17	1	46	
Female	5	39	22	2	68	
Side of injury^c^						0.140^d^
Left	6	33	14	2	55	
Right	1	32	25	1	59	
Extension of the fracture^c^						0.137^d^
Lateral malleolar	1	14	5	1	21	
Bimalleolar M	1	4	0	0	5	
Bimalleolar P	0	11	12	1	24	
Trimalleolar	5	36	22	1	64	
Anterior fracture height (mm)^a^	−4.44 ± 8.86	−7.40 ± 4.30	−4.81 ± 3.58	−3.12 ± 5.10	−6.22 ± 4.62	0.002^e^
Posterior fracture height (mm)^a^	26.82 ± 8.33	23.19 ± 7.55	32.85 ± 15.35	42.76 ± 23.74	27.23 ± 12.32	0.001^b^
Vertical fracture height (mm)^a^	31.26 ± 12.25	30.60 ± 8.29	37.66 ± 14.73	45.88 ± 18.90	33.45 ± 11.89	0.032^b^
Fracture inclination angle (°)^a^	58.00 ± 10.97	55.13 ± 8.47	58.74 ± 10.47	66.79 ± 11.15	56.85 ± 9.58	0.053^b^
Spiral angle of the fracture (°)^a^	238.49 ± 52.29	264.05 ± 34.90	282.58 ± 32.88	301.75 ± 28.25	269.81 ± 37.09	0.009^e^
Spiral angle of the spike (°)^a^	126.94 ± 22.04	150.87 ± 20.55	169.53 ± 22.85	166.78 ± 14.32	156.20 ± 24.04	0.000^e^

^a^ Data presented as the mean ± SD (standard deviation).

^c^ Data presented as no. of cases. The *p*-value is the result of the comparison between type II and type III fractures.

^b^ The result of the Mann‒Whitney *U* test.

^e^ The result of the *t*-test.

^d^ The result of the chi-squared test.

A total of 73 cases (64.0%) had sharp fracture spikes. According to the ratio of the spike height to its base width, there were 38 cases with a long sharp spike pattern and 35 cases with a short sharp spike pattern. Long spike fractures were younger than short spike fractures (*p* = 0.047). Twenty-four cases (24/38, 63.2%) with long spikes had their proximal end-tip apexes on the posterior ridge, while 26 cases (26/35, 74.3%) with short spikes had their apexes on the posterolateral surface (*p* = 0.002). The parameters in the long spike fracture pattern, such as the fracture height in the anterior, posterior, and total directions and the fracture inclination angle, were greater than those in the short spike pattern (Table 4).

**TABLE 4:** Comparison between fractures with different spike patterns.

	Fractures with sharp spike		Fractures with blunt spike	*p*-value^a^	*p*-value^b^
	Long sharp spike	Short sharp spike			
No. of cases (%)	38 (33.3%)	35 (30.7%)	41 (36.0%)		
Patient age (yr)^a^	55.1 ± 14.5	60.5 ± 13.7	61.6 ± 13.7	0.047^*^	0.135^*^
Sex^b^				0.903^e^	0.009^e^
Male	19	17	10		
Female	19	18	31		
Side of injury^b^				0.714^e^	0.932^e^
Left	19	16	20		
Right	19	19	21		
Extension of the fracture^b^				0.561^e^	0.373^e^
Lateral malleolar	4	8	9		
Bimalleolar M	1	1	3		
Bimalleolar P	8	6	10		
Trimalleolar	25	20	19		
Fracture apex distribution^b^				0.002^e^	0.001^e^
Zone I	1	0	6		
Zone II	11	26	28		
Zone III	24	8	7		
Zone IV	2	1	0		
Anterior fracture height (mm)^a^	−4.43 ± 4.87	−7.09 ± 4.45	−7.13 ± 4.12	0.021^c^	0.138^c^
Posterior fracture height (mm)^a^	37.73 ± 13.93	22.27 ± 6.85	21.74 ± 7.25	0.000^c^	0.000^c^
Vertical fracture height (mm)^a^	42.16 ± 13.60	29.36 ± 8.05	28.87 ± 8.07	0.000^c^	0.001^c^
Fracture inclination angle (°)^a^	62.49 ± 8.25	53.45 ± 9.62	54.52 ± 8.46	0.000^c^	0.051^d^
Spiral angle of the fracture (°)^a^	284.16 ± 31.05	269.11 ± 33.82	257.11 ± 40.75	0.051^d^	0.006^d^
Spiral angle of the spike (°)^a^	162.63 ± 23.95	158.25 ± 24.59	148.50 ± 22.00	0.444^d^	0.01^d^

^a^ Data presented as the mean ± SD.

^b^ Data presented as no. of cases. The *p*-value^a^ is the result of the comparison between fractures with long spikes and fractures with short spikes. *p*-value^b^ is the result of the comparison between fractures with spikes and fractures without spikes.

^c^ The result of the Mann‒Whitney *U* test.

^d^ The result of the *t*-test.

^e^ The result of the chi-squared test.

The sharp spikes were further broken in 21 cases, most of which were in female patients with trimalleolar fractures. In general, a broken spike was more commonly seen in patients with a longer fracture line, greater fracture inclination angle, and greater fracture spiral angle (Table 5).

**TABLE 5:** Comparison between fractures with or without broken spikes.

	Fractures with or without broken spike		*p*-value
	Broken	Unbroken	
No. of cases (%)	21 (18.4%)	93 (81.6%)	
Patient age (yr)^a^	59.1 ± 14.8	59.1 ± 14.0	0.884^c^
Sex^b^			0.087^c^
Male	5	41	
Female	16	52	
Side of injury^b^			0.303^e^
Left	8	47	
Right	13	46	
Extension of the fracture^b^			0.083^e^
Lateral malleolar	2	19	
Bimalleolar M	0	5	
Bimalleolar P	2	22	
Trimalleolar	17	47	
Spike type^b^			0.074^e^
Sharp spike (long or short)	17 (13/4)	56 (25/31)	0.021^e^
Blunt spike	4	37	
Fracture apex distribution^b^			0.201^e^
Zone I	1	6	
Zone II	8	57	
Zone III	11	28	
Zone IV	1	2	
Anterior fracture height (mm)^a^	−6.32 ± 3.75	−6.19 ± 4.81	0.959^c^
Posterior fracture height (mm)^a^	35.43 ± 15.33	25.38 ± 10.80	0.001^c^
Vertical fracture height (mm)^a^	41.76 ± 14.12	31.58 ± 10.54	0.002^c^
Fracture inclination angle (°)^a^	61.55 ± 9.80	55.79 ± 9.25	0.012^d^
Spiral angle of the fracture (°)^a^	287.45 ± 25.53	265.83 ± 38.22	0.016^c^
Spiral angle of the spike (°)^a^	161.88 ± 24.17	154.82 ± 23.95	0.232^d^

^a^ Data presented as the mean ± SD.

^b^ Data presented as no. of cases.

^c^ The result of the Mann‒Whitney *U* test.

^d^ The result of the *t*-test.

^e^ The result of the chi-squared test.

### 3.2 The distribution of the fracture apex of type B lateral malleolar fractures

According to the distribution of the fracture apex in the 3D model, there were seven cases in zone I (lateral ridge), 65 cases (57%) in zone II (posterolateral surface), 39 cases (34.2%) in zone III (posterior ridge), and three cases in zone IV (medial surface). In total, 49 cases (43%) had their fracture apexes not on the posterolateral surface of the fibula, as 39 cases (34.2%) had their apexes on the posterior ridge. Patients with zone II fracture apex distribution (mean 63.6 years) were significantly older than those with zone III (mean 52.7 years) (*p* = 0.000), as 47 cases (72.3%) in zone II were older than 60 years (Table.3).

### 3.3 3D fracture line map of type B lateral malleolar fractures

**FIGURE 7:**
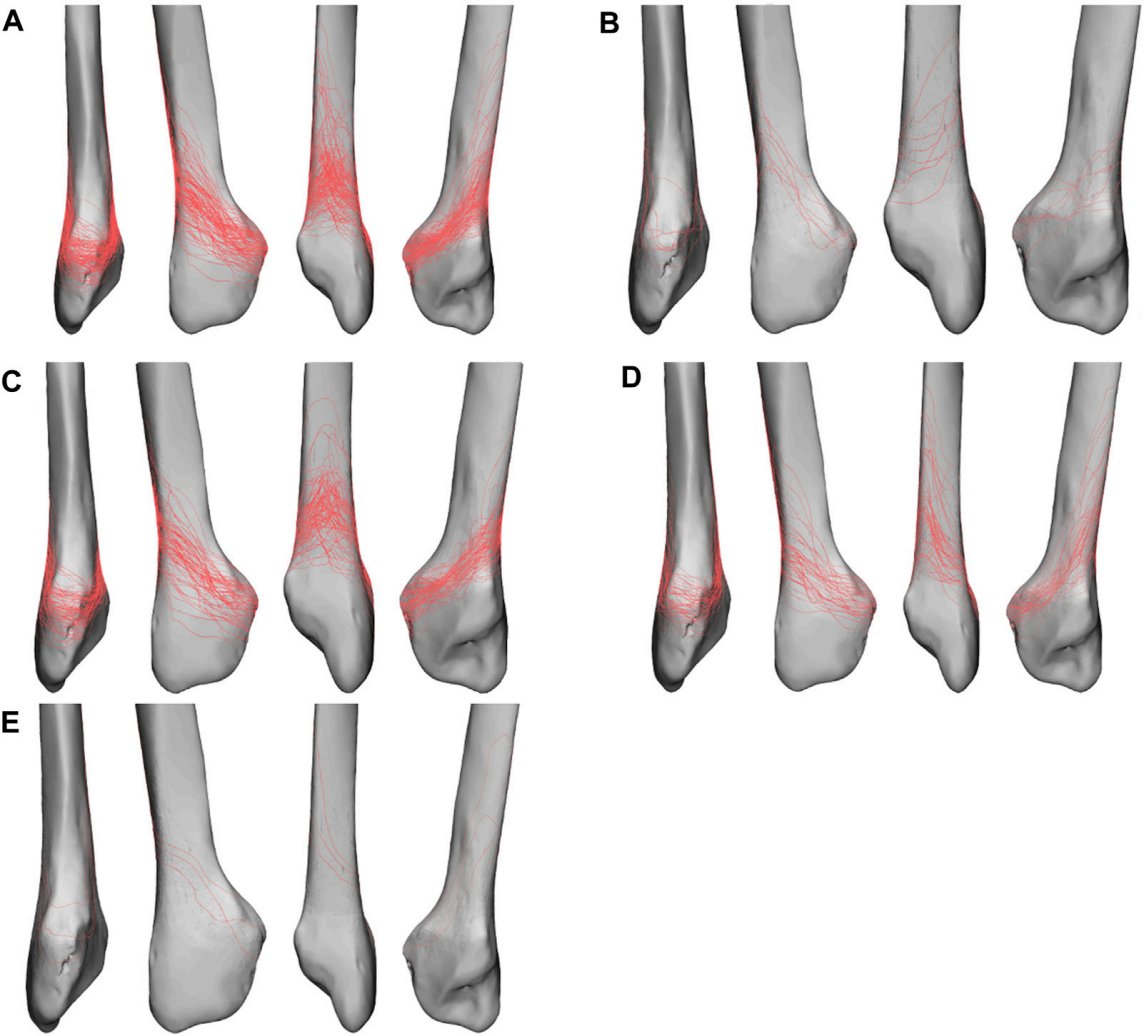
Fracture line maps in anterior, lateral, posterior, and medial views. **(A)** Fracture line map of all fractures. **(B)** Fracture line map of zone I apex fractures. **(C)** Fracture line map of zone II apex fractures. **(D)** Fracture line map of zone III apex fractures. **(E)** Fracture line map of zone IV apex fractures.

The 3D fracture map showed that most of the fracture lines in type B lateral malleolar fractures originated from the anterior tuberosity of the fibula, which was the insertion of the anterior tibiofibular ligament, and ran laterally backward and upward. The fracture lines are relatively concentrated on the anterior, lateral, and medial sides but diverse on the posterior side. Compared to the zone II fracture apex, the fracture lines of the zone III apex were higher on the anterior view and steeper on the lateral, medial, and posterior views (Figure 7).

## 4 Discussion

Type B lateral malleolar fractures caused by low-energy trauma are increasing with the aging of the population (Juto et al., 2018). In our study, 68 (59.6%) patients were female, with 70.6% of them ≥60 years old, and female patients accounted for 67.2% of trimalleolar fractures. These findings are consistent with the research of Vieira Cardoso et al. (2021), who reported that female and elderly patients are more likely to suffer trimalleolar fractures. Postmenopause and aging are risk factors for osteopenia, which leads to reduced bone quality and brings technical challenges to the stable fixation of the fracture (Hsu et al., 2019).

### 4.1 Spiral features of type B lateral malleolar fractures

Spiral pattern fractures in humans are commonly seen in the humeral, femoral, and tibial shafts. Spiral fractures are caused by torsional forces but may be combined with compression and bending vectors. Through an experimental biomechanical study, VanCourt et al. (2000) found that if torsional force alone was applied, the fracture morphology showed that the fracture torn length was similar to the diameter of the bone and the inclination angle was approximately 45°. The fracture length was greater than the diameter of the bone if a longitudinal compression force was applied, and a third butterfly fragment was observed on the stressed side if a bending force was applied. Ankle fractures are usually caused by lateral rotational force, so most lateral malleolar fractures demonstrate a spiral fracture fashion.

In our study, when considering the total fracture height and the fracture spiral angle, we found that these parameters were greater in fractures with sharp spikes than in those with blunt spikes and, moreover, in fractures with long sharp spikes than in those with short sharp spikes and in zone III apex fractures than in zone II apex fractures. It can be inferred that the torsional and compression forces suffered by the former are greater than those suffered by the latter. The proportion of fractures with sharp spikes in male patients was as high as 78.3%, while the majority (75.6%) of female patients had fractures with blunt spikes, suggesting that the injury violence of ankle fractures in males is greater than that in females, which is consistent with the findings of Juto et al. (2018).

Twenty-one patients in our study had their spikes further broken, most of whom were females, with trimalleolar fractures and fractures with sharp spikes. The total fracture height and fracture spiral angle with broken spikes were greater than those without broken spikes. Refracture of the spike indicates its suffering from bending force along with torsional and longitudinal compression.

The location of the end-tip apex and refracture of the spike reflect the characteristics of the violence suffered at the time of injury, which may have an influence on the decision of treatment protocol. These characteristics may also be related to the incidence and fracture patterns of the posterior and/or medial malleolus.

### 4.2 The relationship between the morphology of type B lateral malleolar fractures and tibiofibular syndesmotic stability

In recent years, many studies have explored the relationship between the morphology of type B lateral malleolar fractures and tibiofibular syndesmotic stability (Choi et al., 2014; Chun et al., 2019; Kellam et al., 2021; Cao et al., 2022). Choi et al. (2014) found that young male patients with high-energy injuries were more prone to suffer tibiofibular syndesmotic instability; in terms of fracture morphology, the higher the anterior fracture line, the larger the medial clear space, and the better the bone quality, the more likely the ankle fracture will be accompanied by syndesmotic instability. Chun et al. (2019) found that fractures with long and pointed distal bone fragments are more likely to be syndesmotic unstable. Cao et al. (2022) used 3D fracture line map technology and found that when the fracture line was high and steep, it usually indicated that the tibiofibular syndesmosis was unstable. They also found that the fracture height and the fracture inclination angle were important predictors of syndesmotic instability; that is, the greater the fracture height and inclination angle, the greater the possibility of syndesmotic instability.

Our study found that the anterior fracture origin and posterior fracture termination in zone III apex distribution fractures were significantly greater than those in zone II apex fractures. More than 60% of zone III apex fractures were long sharp spike fractures, while more than 80% of zone II apex fractures had short sharp spikes or blunt spikes. The 3D fracture line map also showed that the fracture lines of the zone III apex were steeper than those of zone II. These findings may imply that lateral malleolar fractures with a zone III apex distribution are more prone to syndesmotic instability.

### 4.3 Plate fixation of lateral malleolar fractures

Type B lateral malleolar fractures are usually fixed with a lateral neutralization plate or a posterolateral antiglide plate. The posterolateral antiglide plate has been shown to have better biomechanical stability than the lateral neutralization plate, which is more evident in the fixation of osteoporotic fractures (Schaffer and Manoli, 1987; Minihane et al., 2006). With the advent of locking plates, various types of lateral locking plates have been used for lateral malleolar fractures (Schepers et al., 2011). Biomechanical studies confirmed that it has similar or even better stability than the posterolateral antiglide plate (Hallbauer et al., 2014; Switaj et al., 2016). However, a thicker locking plate may result in an increased incidence of wound dehiscence (Schepers et al., 2011). The skin and soft tissue coverage on the lateral side of the lateral malleolus is fragile, and placing a thick locking plate on the lateral side may cause skin irritation and increase the occurrence of postoperative plate-related pain, thereby impairing ankle function (Brown et al., 2001). Several biomechanical studies have found that fixation failure after locking plates often results in comminution of the distal bone fragment, causing difficulties in subsequent revision. However, failure after antiglide plate fixation is often caused by bending of the plate with the pullout of distal screws, and the distal fragment is relatively intact (Schaffer and Manoli, 1987; Minihane et al., 2006; Switaj et al., 2016).

**FIGURE 8:**
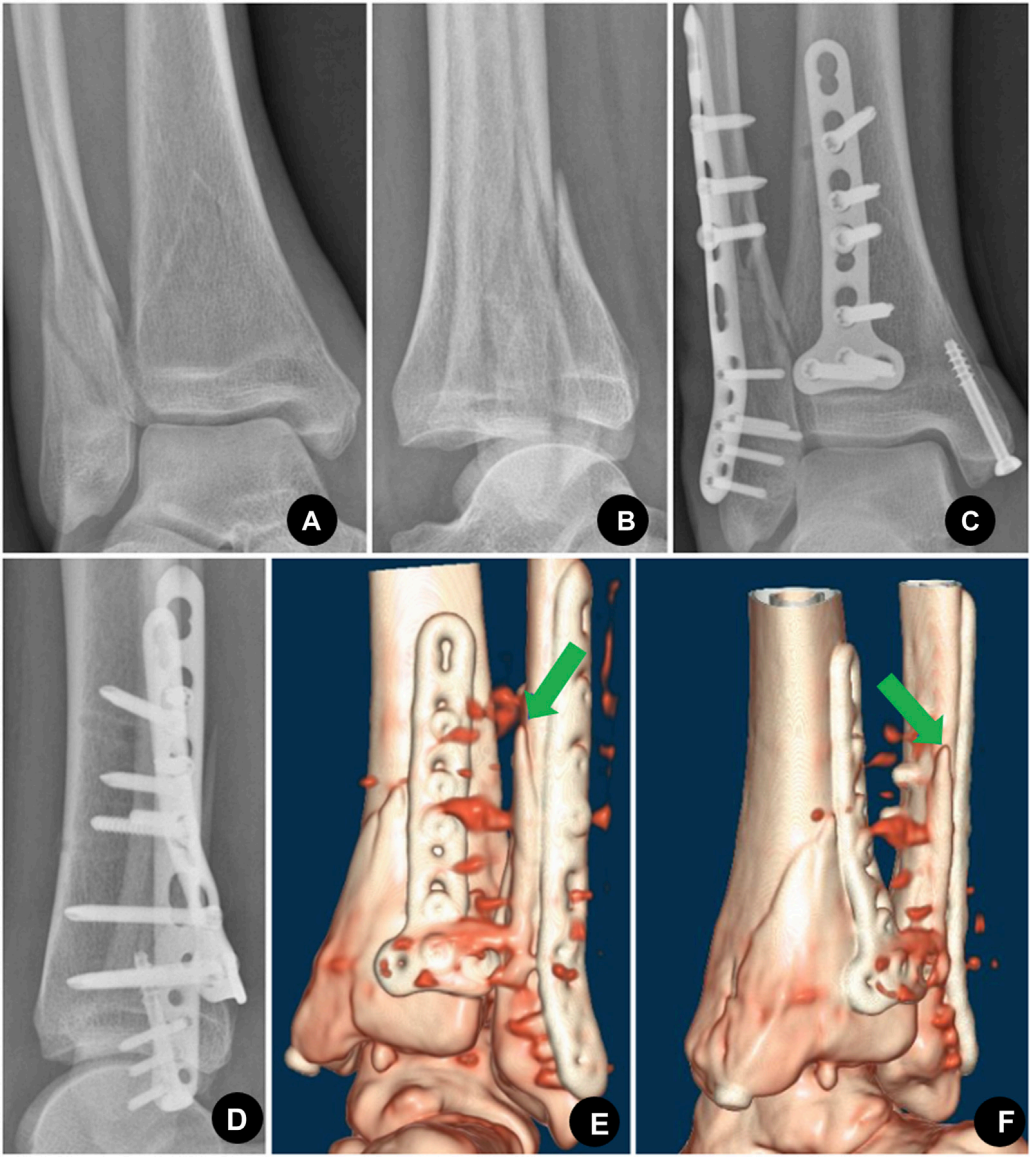
Sixty-seven-year-old female with trimalleolar fractures, the proximal apex of the lateral malleolus was located in zone III. **(A)**, **(B)** Preoperative AP (antero-posterior) and lateral X-ray showed trimalleolar fractures with posterior dislocation of the ankle. **(C)**, **(D)** Postoperative AP and lateral X-ray showed that the lateral malleolar fracture was fixed with the posterolateral antiglide-buttress plate, the medial malleolar was fixed with a cannulated compression screw, and the posterior malleolar was fixed with a T-shaped plate. **(E)**, **(F)** Posterior and posteromedial view of the postoperative CT volume rendering showed that the distal fibular fracture apex was not compressed by the posterolateral plate as the apex (indicated by a green arrow in E and F) was located on the posterior ridge (zone III) which could not be reached by the pre-existing plate. As a result, the stability of the construct was impaired.

Our study found that 77% of type B lateral malleolar fractures occurred as trimalleolar or bimalleolar fractures. Lateral and posterior malleolar fractures are usually treated through a single posterolateral approach (Little et al., 2013). Using an antiglide plate to fix lateral malleolar fractures through the posterolateral approach is more convenient and requires less dissection. For these reasons, the application of antiglide plates is becoming increasingly popular. Several clinical studies have demonstrated its good outcome and low incidence of peroneal tendonitis with proper application, which included avoiding screws in the most distal hole or sinking the screw wholly into the plate (Wenger et al., 2021; DeKeyser et al., 2022). The key to obtaining an antiglide function is to compress over the fracture apex by an undercontoured plate. If a plate cannot compress over the fracture apex, it is called a buttress plate, not an antiglide-buttress plate. Our study demonstrated that 43% of type B lateral malleolar fractures’ end-tip apexes were not on the posterolateral surface, as 34.2% were on the posterior ridge. These fracture apexes could not be compressed by the currently available plates, which reduce the stability of fracture fixation (Figure 8). This should also be considered in future biomechanical studies, especially in studies comparing fixation stabilities. The location of the fracture apex may have a significant impact on fixation stability.

There are some limitations of this study. First, this is a retrospective study. We failed to record the cause of the patient’s injury in detail, which made it impossible for us to analyze the relationship between the location of the fracture apex and the cause of the injury. However, we inferred the type of violence from the shape of the fractures. Second, some patients were not included in the study for various reasons, which may bias our research results. However, the baseline data of our cases were similar to those of previous studies (Vieira Cardoso et al., 2021). Third, we did not conduct interobserver and intraobserver validation, which may have impaired the results. However, the types of end-tip location and the parameters for measuring were all jointly discussed by the two authors. Finally, there were not enough zone I and zone IV fractures for comparison in our study. Although we have shown the morphological differences between zone II and zone III fractures, examining more cases may enable us to more accurately reflect the relation between the location of the fracture apex and fracture morphology and injury mechanism.

In summary, nearly half of the proximal fracture apexes in type B lateral malleolar fractures were not on the posterior surface, which may not be compressed by the currently available plates in the antiglide mode. Further improvement in plate design is needed. The steeper the fracture line and the longer the fracture spike, the greater the posteromedial fracture apex distribution will be, which indicates more severe violence and accompanying injuries. The relationship between fracture apex distribution and the morphology of the posterior and medial malleolus, which may also have a significant impact on clinical practice, needs further investigation.

## Data Availability

The original contributions presented in the study are included in the article/Supplementary Material; further inquiries can be directed to the corresponding author.
